# Retraction Note: Structure based innovative approach to analyze aptaprobe–GPC3 complexes in hepatocellular carcinoma

**DOI:** 10.1186/s12951-025-03221-4

**Published:** 2025-02-18

**Authors:** Woo-Ri Shin, Dae-Young Park, Ji Hun Kim, Jin-Pyo Lee, Nguyen Quang Thai, In-Hwan Oh, Simranjeet Singh Sekhon, Wooil Choi, Sung Yeon Kim, Byung-Kwan Cho, Sun Chang Kim, Jiho Min, Ji-Young Ahn, Yang-Hoon Kim

**Affiliations:** 1 School of Biological Sciences, Chungbuk National University, 1 Chungdae-Ro, Seowon-Gu, Cheongju, 28644 Republic of Korea; 2 Department of Biological Sciences, Korea Advanced Institute of Science and Technology, 291 Daehak-ro, Yuseong-gu, Daejeon, 34141 Republic of Korea; 3 Graduate School of Semiconductor and Chemical Engineering, Jeonbuk National University, Jeonju, 54896 Republic of Korea; 4 College of Pharmacy, Wonkwang University, Shinyoung-dong 344-2, Iksan, Jeonbuk 570-749 Republic of Korea

**Retraction Note: Journal of Nanobiotechnology (2022) 20:204**

10.1186/s12951-022-01391-z

The Editor-in-Chief has retracted this article. After publication, concerns were raised regarding Fig. 5a, specifically:

- The Hep3B 0 pM image appears highly similar to PANC-1 100 pM;
- The HeLa 200 pM and MIA-PaCa 50 pM images appear to overlap;
- The PANC-1 50 pM image appears highly similar to MIA-PaCa 100 pM.

In addition, in Fig. 6a, the GPC3_3 30 and 120 min mice appear highly similar, with identical leg and ear positions, but different fluorescence.

The authors have provided partial raw data to address these concerns. However, the Publisher has been unable to verify the authenticity of these data, and found further similarities in the original images from the experiment presented in Fig. 6a. The Editor-in-Chief therefore no longer has confidence in the presented data.

Ji-Young Ahn has stated on behalf of all authors that they do not agree to this retraction.

